# Driver Gene Alterations in Malignant Progression of Gastric Cancer

**DOI:** 10.3389/fonc.2022.920207

**Published:** 2022-07-12

**Authors:** Yuanqiang Dong, Ning Song, Jun Wang, Liubin Shi, Ziqiang Zhang, Jianjun Du

**Affiliations:** Department of General Surgery, Huashan Hospital, Fudan University, Shanghai, China

**Keywords:** gastric cancer, driver gene, mutation, immunity, prognosis

## Abstract

The identification of driver genes is of great importance in modern medical research. It is also an essential factor in the development of individualization and has a positive effect on understanding the causes of cancer. Gene mutations are the primary cause of the outcomes of the process of tumorigenesis. Driver genes can be used as therapeutic targets for tumor-specific mutation-dependent overexpression. This study sought to identify mutation-based driver genes in gastric cancer (GC) by applying comprehensive gene expression and copy number analysis. Multiplatform analysis was used to identify four major genomic subtypes of GC. The most prominent cancer-related variations observed in this cohort were *TTN* mutations (found in 56% of tumors), followed by *TP53* (51%), *MUC16* (7%), and *LRP1B* (6%) mutations. In our analysis, mutation characteristics were mainly related to the DNA mismatch repair system. In addition, 34 candidate driver oncogenes were identified in GC. Further research identified six GC-related driver genes associated with the levels of immune infiltration of different immune cells and the majority of immune markers. Our mutation-based study of driver oncogenes identified potential drug targets in GC.

## Introduction

Gastric cancer (GC) is the third leading cause of cancer-related death worldwide (1). GC includes a group of heterogeneous diseases with various pathological, genetic, and cellular properties. Since the clinical symptoms of GC generally appear late in its clinical course, there are constantly smaller numbers of options for surgical therapy, making diagnoses of early GC difficult (2). Due to the low overall survival rates of patients with GC (especially those with metastatic disease), new treatments for GC are urgently needed.

Different classification systems can be used in medicine for the histological classification of GC. The Lauren classification and World Health Organization classification are the two most widely accepted systems. Nevertheless, it should be noted that while the existing histopathological framework occasionally affects endoscopic or surgical approaches, the precise treatment of individual patients remains lacking (3).

Therefore, alternative classification systems are required. Fortunately, emerging scientific and technological achievements have enabled researchers to conduct high-resolution molecular research on GC following the continued improvement of genome and high-performance analysis technologies. Such molecular data make it possible to identify candidate driver modifications in GC, including genetic mutations, chromosomal changes, transcription changes, and epigenetic disorders. Understanding potential changes in the pathogenesis of GC may also contribute to the discovery of clinically significant biomarkers and potential therapeutic goals. The TOGA research demonstrated that the overall survival rate of patients with Her2/neu-positive GC could be enhanced by following treatment with trastuzumab in combination with chemotherapy (4).

The Cancer Genome Atlas (TCGA) research team recently investigated the molecular mechanism of each GC subgroup, to fully understand GC at the molecular level and explained it for each GC subgroup (5). Although no identifiable genes or oncogenes were discovered in the majority of cases of GC in the TCGA, transcription and epigenetic analysis identified subtypes of the disease and were considered to represent the downstream effects of carcinogenesis (5). Only a small number of patients with GC represented in the TCGA were investigated in the original GC study led by the TCGA research team, and data were generated in other cases following an earlier study. With the latest TCGA data generation stage results, a systematic analysis of the TCGA STAD cohort is possible. This allows to compare and contrast various cancers and provide a wide dataset to further improve the capability of detecting important molecular processes. Thus, we aimed to identify driver gene alterations and their correlation with immune infiltration levels and the subsequent signaling pathways that drive the progression of GC to develop more effective therapies.

## Materials and Methods

### TCGA Datasets

We used the R package, TCGAbiolinks, to download the mutant MAF file for GC (this MAF file’s reference genome is hg19). This MAF file contains the mutation detection results for 439 samples. For the 393 cases of GC obtained from FireBrowse, methylation chip data were downloaded. In addition, data from 439 cases of GC were downloaded from the SNP6 copy number fragment (http://firebrowse.org/). Simultaneously, we obtained 375 GC specimens with mRNA expression profile data and 436 miRNA expression profile information from the available GDC data (https://portal.gdc.cancer.gov/). In the above data, 332 samples had multi-omics data, including data regarding mutations, copy number variation (CNV), methylation, and expression profile for mRNA and miRNA. The basis for the subsequent study was 332 samples.

### Driver Gene Analysis

MutSigCV, which screens for genes with increased mutation frequency, more mutations, and more mutations in conserved areas, is an effective way to screen for high-frequency tumor mutants. Genomes leave different markers when mutations occur. At present, analysis has revealed more than 30 mutation patterns. Here, we used the R package, maftools, and somatic signatures to analyze the mutation status of tumor samples and draw the mutation spectrum and mutation characteristics.

### CNV Analysis

The genomic identification of significant targets in cancer (GISTIC) algorithm is used to detect the copy number change region common in all samples, including the chromosomal arm-level CNVs and the smallest common region between the samples. The GISTIC algorithm parameters were set as Q ≤ 0.05, to define a significant change; when determining the peak interval, the confidence level was 0.95; when analyzing chromosomal arm-level variation, a region greater than a chromosome arm length of 0.98, was set as the standard. The R package, ABSOLUTE, was used to analyze the pure tumor and the ploidy study based on the CNV findings.

### Quantitative Real-Time Polymerase Chain Reaction

Bio-Rad quantitative PCR system were used to perform qRT-PCR (Hercules, CA, USA). The results were normalized to β-actin for mRNA measurement. Counts are reported as fold change relative to the normal control. All the primers are listed in **Supplementary Table 1**. Each experiment was repeated three times on each condition.

### Discovery of Multiplatform-Based Subtypes

GC specimens were subtyped according to as per the different DNA methylation data platforms, changes in DNA copying, mRNA expression, and miRNA expression, as characterized by the additional methods. The subtypes characterized at each site were coded for each subspecies as a series of indicator variables. To evaluate the integrated subspecies. Matrices 1 and 0 underwent cluster-of-clusters analysis (COCA) (6, 7). The analysis comprised of initial two-way t-tests for each gene, comparing each subspecies with the remainder of the tumor, and then screen the top 100 genes with the lowest p-value in each subspecies.

## Results

### Somatic Genomic Alterations in GC

**Figure 1:**
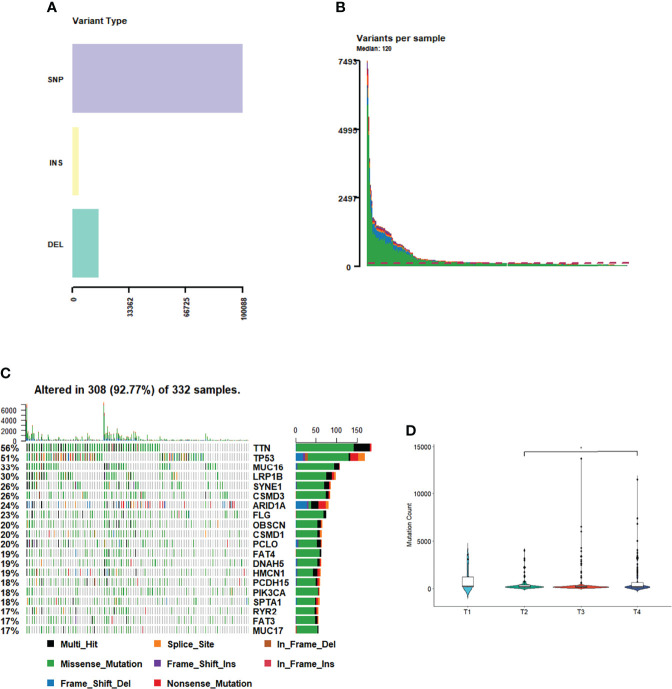
Tumor mutation profile. **(A)** Variant type of GC. **(B)** Variants in each sample. **(C)** The most mutations in GC. **(D)** The number of mutations in T phases of GC.

Among the GC samples, 130,936 somatic mutations were identified, comprising 110,054 single-nucleotide variants and 20882 insertions or deletions (INDELs) (**Figure 1A**). These tumors harbored a median of 120 variants (**Figure 1B**). As demonstrated in **Figures 1A, B**, the most prominent cancer-related variations observed in this cohort were *TTN* mutations (found in 56% of tumors), followed by *TP53* (51%), *MUC16* (7%), and *LRP1B* (6%) mutations (**Figure 1C**). We separately calculated the correlation between the number of somatic mutations and the clinical features of each tumor sample. The results showed that the number of mutations in the T4 phase was significantly higher than that in the T2 phase (**Figure 1D**). However, the number of somatic mutations and the tumor phase or tumor survival status were no strongly correlated (**Supplementary Figures 1A–E**), which may suggest that holistic analysis of mutations and clinical features of the GC samples could only find some clinical features with a strong correlation to mutations. However, through further molecular classification, it could be observed that different types of samples may have significant differences in some clinical features.

### Mutation Spectrum analysis

**Figure 2:**
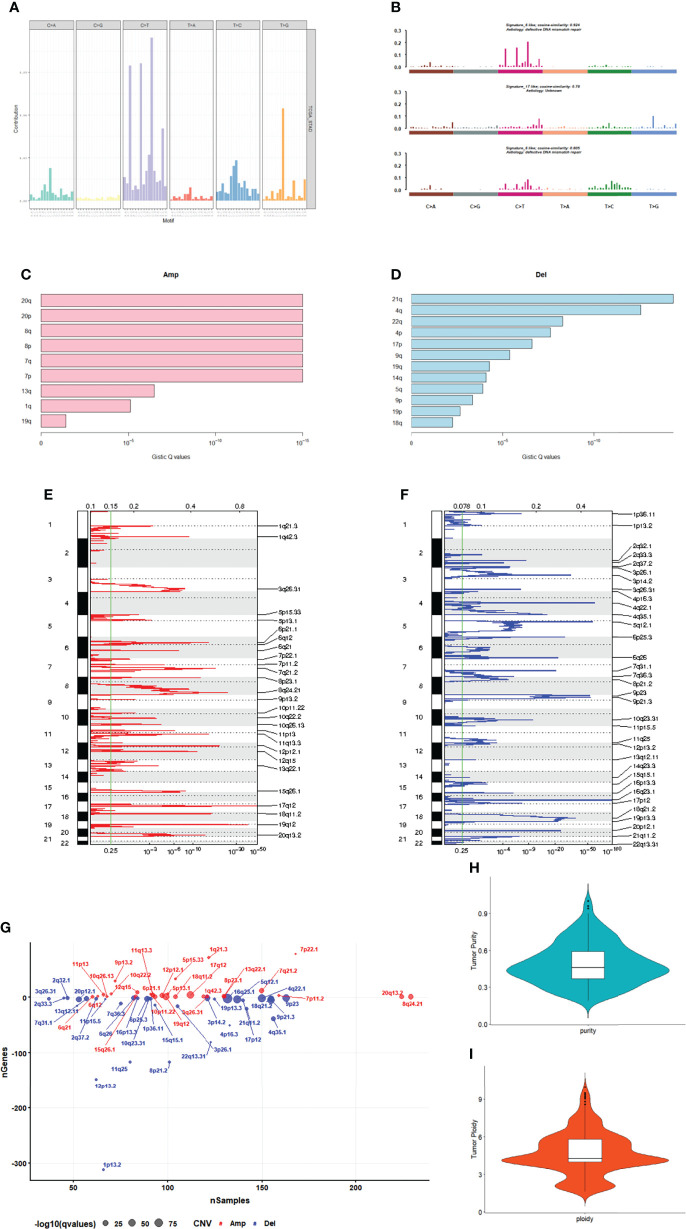
Mutation and CNV analysis. **(A)** Mutation spectrum distribution. **(B)** The similarity between mutation features and cosmic mutation signature. **(C)** Amplification in chromosomal arms. **(D)** Deletion in chromosomal arms. **(E)** The distribution of CNVs amplificated regions. **(F)** The distribution of CNVs deleted regions. **(G)** The number of genes in CNVs amplificated or deleted regions. **(H)** The purity range of GC. **(I)** Tumor cell ploidy of GC.

A number of mutation types, including C>A, C>G, C>T, T>A, T>C, and T>G, occur in cancer. There are four possibilities for each base considering the upstream 1 bp and downstream 1 bp: A, T, C, G; therefore, we classified the mutations into 96 different types based on the context (**Figure 2A**). We analyzed the patterns of base changes within gastric cancer tumors and noted elevated rates of C to T transitions as previously observed (5).To investigate the link between GC sample mutation frequency allocation and COSMIC database signature, three types of somatic mutations were extracted by conducting non-negative matrix factorization on a frequency matrix with 332-row specimens and 96 columns of mutation types. The analysis showed that signature 6 was mainly associated with the GC spectrum of mutations because of the lack of DNA mismatch repair and signature 17 (**Figure 2B**).

### CNV Analysis

GC samples were analyzed using the GISTIC software. Among them, 20q, 20p, 8q and 8p amplification (**Figure 2C**) and 21q, 4q and 22q deletions (**Figure 2D**) were the most significant. A total of 27 amplified copies and 34 copy number deletions were detected among all tumor samples. Among the detected concentrated CNVs, the most significantly amplified regions were 17q12 and 19q12 (**Figures 2E, G**). Meanwhile, the most significant deleted regions were 16q23.1 and 4q22.1 (**Figures 2F, G**). We performed tumor purity and ploidy assessment of the samples based on the CNV information for each tumor sample. The purity range of the tumors was 0.19 to 1 (**Figure 2H**), and the tumor cell ploidy was 1.61 to 9.97 (**Figure 2I**), indicating that genomic abnormality is indeed a normal phenomenon in tumor-genesis.

### Four Major Genomic Subtypes of GC in Multiplatform Analysis

**Figure 3:**
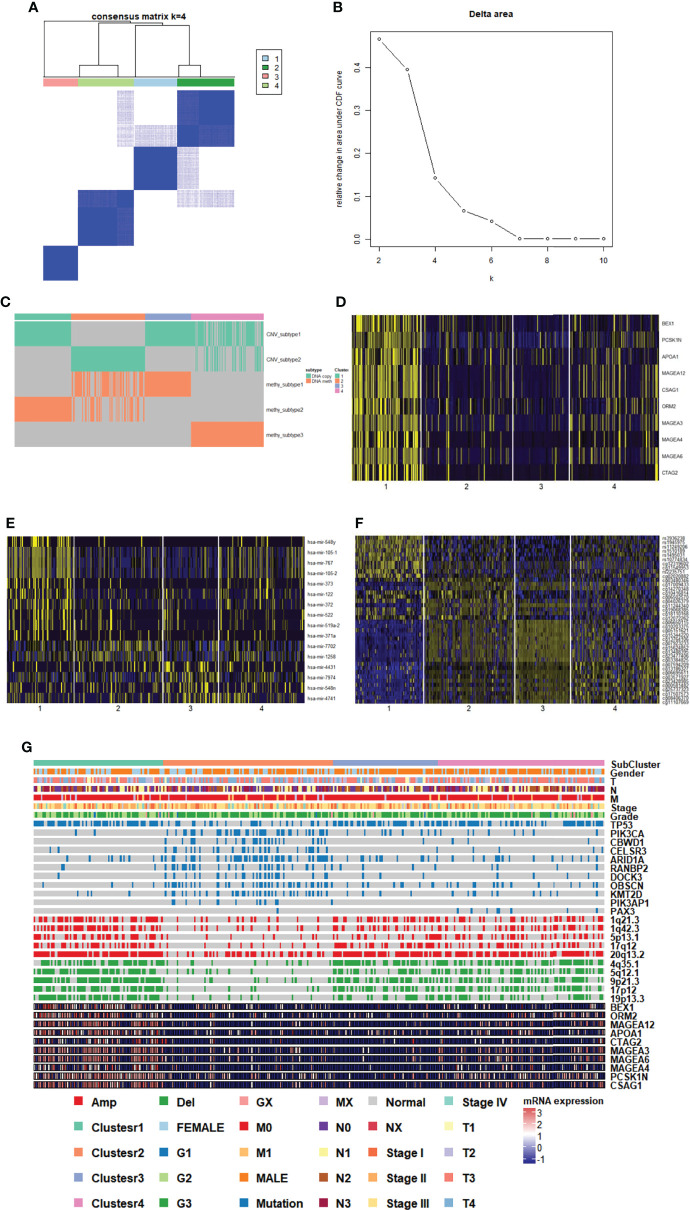
Genomic subtypes of GC in multiplatform analysis. **(A, B)** The result of clustering analysis based on the multiple platforms. **(C)** Subgroup distribution and clustering analysis base on single platform. **(D)** Heatmap of subgroup specific genes. **(E)** Heatmap of subgroup specific miRNAs. **(F)** Heatmap of subgroup specific methylation sites. **(G)** Subgroup features integrated landscape map.

We characterized GC samples using four sets of molecular data: CNV, methylated expression profile, mRNA expression profile, and miRNA expression profile (**Supplementary Figures 2A–D**). Since the clustering effect of mRNA and miRNA expression profiles was not significant, CoCA was used to perform the re-clustering analysis based on the CNV and methylation platform subgroup classification results. The most durable categorization was achieved with four categories (**Figures 3A–C**).

To provide a general description of the clinical and molecular characteristics of GC subpopulations, we showed the distribution of clinical features of each sample and the expression, mutations, and CNV of partial genes in each subpopulation. We could clearly see that cluster 1 had high-expressing characteristic genes (including MAGEA12), and cluster 2 had more mutations and fewer CNV. Clusters 3 and 4 belonged to subgroups with fewer mutations and more CNVs (**Figure 3G**). The association between each cluster and clinical characteristics, along with cancer type and differentiation, were further analyzed. The G2 and G3 sample distributions were significantly different among subgroups (p=0.0278), and the G3 samples in cluster 2 and cluster 4 were much higher than the in G2 samples, indicating that the samples in the two clusters were more malignant (**Figure 3G**).

To identify the characteristic genes in each subgroup, we calculated the differentially expressed genes and miRNAs in each subgroup. We screened the top 10 genes (**Figure 3D**, **Supplementary DataSheet 1**) and miRNAs in the subgroup (**Figure 3E**, **Supplementary DataSheet 2**). At the same time, we evaluated the methylation sites of areas of differential expression in each subgroup (**Figure 3F**, **Supplementary DataSheet 4**). In the case of specific expression gene analysis, only genes highly expressed in cluster 1 were found, and the gene expression differences among the other three clusters were not significant, suggesting that cluster 1 may vary dramatically from other genetic expression subsets.

### Driver Gene Detection and Prediction

A large number of somatic cell mutations occur during the development of cancer, among which a small number of genes called driver genes could directly affect the occurrence and development of tumors. Driver gene prediction was performed using MutSigCV for mutation data from tumor specimens. Researchers screened 34 candidate driver genes in the range of importance with a q threshold<0.01 (**Supplementary DataSheet 2**), and three of the candidate genes were among the top20 most mutated. These genes were *TP53*, *ARID1A*, and *PIK3CA*. The researchers identified previously reported genes (*TP53*, *ARID1A*, and *CDH1*) and new driver genes with substantial mutations (*PIK3CA*, *CDC27*, *CTCF* and *IL12* among others). We further detected their mRNA expression with PCR and found significant difference in *ACVR2A*, *CDC27*, *CDH1*, *CDKN2A*, *CTCF*, *IL32*, *ALRP4B*, *NUDT11*, *POLDIP2*, *PTEN*, *PTH2* and *RHOA* between tumor and normal tissue (**Supplementary Figure 3**).

### Gene Mutation in Subgroups

**Figure 4:**
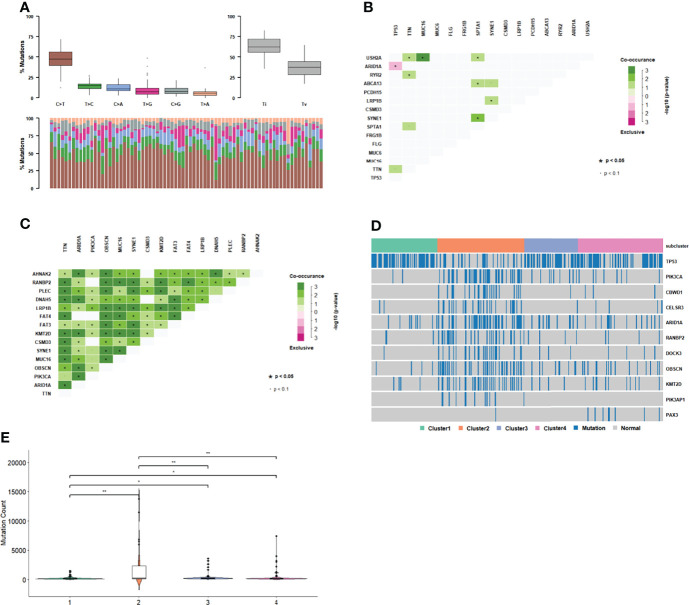
Gene mutation in subgroups. **(A)** Mutation type distribution in cluster 1. **(B)** Co-occurrence and mutual exclusivity of genetic alterations in cluster 1. **(C)** Co-occurrence and mutual exclusivity of genetic alterations in cluster 2. **(D)** Gene mutation distribution in subgroups. **(E)** Distribution of mutation number among subgroups.

The gene mutations in each cluster were divided into two categories: transition and transversion. We determined whether each cluster differed in the type of mutation. Each cluster had C>T mutations, and the transition ratio was found to be usually greater than the transversion ratio (**Figure 4A**, **Supplementary Figures 4A–C**). Co-occurrence or mutual exclusivity of genetic alterations are often observed in cancer. Among them, *TP53* and *ARID1A* were found to be exclusive mutations in cluster 1 (**Figure 4B**), and more genes in cluster 2 were co-mutated (**Figure 4C**, **Supplementary Figures 4D, E**).

There were 1226 genes with different mutations in different clusters (p<0.01, **Figure 4D**). The number of mutations in cluster 2 was significantly higher than that in the other three clusters (**Figure 4E**). *TP53* mutations, which are common in GC samples, were less common in cluster 2 than in the other clusters. In cluster 2, there was a significant exclusive mutation of *TP53*, which is *ARID1A*. These results indicate that cluster2 had molecular features that were significantly different from those of the other clusters.

**Figure 5:**
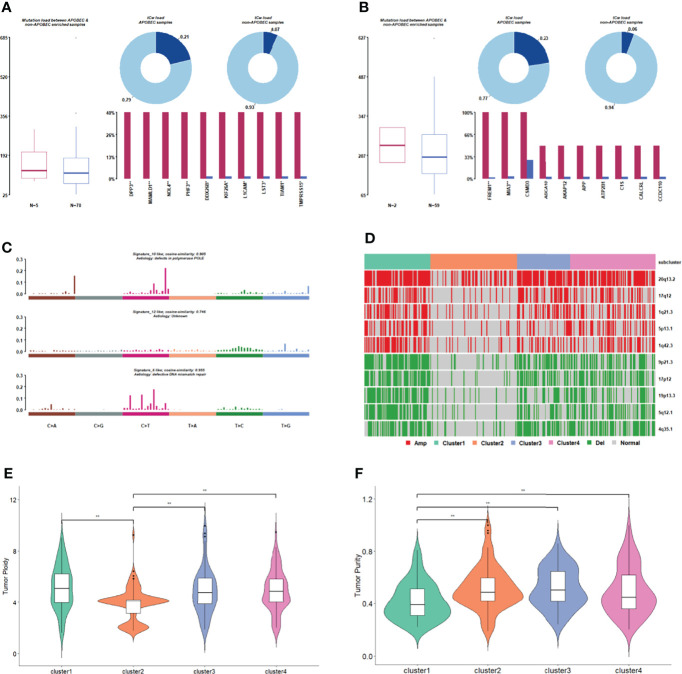
Comparison of gene mutation in subgroups. **(A)** APOBEC enrichment analysis in cluster 1. **(B)** APOBEC enrichment analysis in cluster 3. **(C)** The signature with high degree similarity in cluster 2 and cosmic signature. **(D)** The distribution of CNV regions with significant differences in amplified/deleted regions among subgroups. **(E)** Tumor ploidy distribution among subgroups. **(F)** Tumor purity distribution among subgroups.

Apolipoprotein B mRNA-editing enzyme catalytic polypeptide (APOBEC) is an evolutionarily conserved cytidine deaminase group. Different homologous catalytic subunits can edit RNA or DNA to catalyze the deamination of cytosine into urine pyrimidine or thymine. The genes in APOBEC-enriched samples with significantly higher mutation ratios are shown in **Figures 5A, B**. Most samples in each subgroup were non-APOBEC enriched samples. The APOBEC enriched samples were not found in cluster 2 or 4.

We then counted the 96 mutation types of each cluster, performed signature analysis, and compared them with the signatures included in the Cosmic database (**Figure 5C**, **Supplementary Figures 5A–C**). Signature10 and signature12 were specifically found in cluster 2 (**Figure 5C**), and signature10 was related to polymerase defects. Signature15 was specifically found in cluster 3 (**Supplementary Figure 5B**), which is related to DNA mismatch repair. Signature21 and signature3 were specifically found in cluster 4 (**Supplementary Figure 5C**), and signature3 was related to DNA-DSB repair defects.

A copy number shift analysis of all chromosome segments for every subcategory identified 34 missing regions and 23 amplified areas (**Figure 5D**). The proportion of samples with copy number changes in cluster 2 was significantly lower than that in the other three clusters. The tumorigenesis in cluster 2 appears to be mainly related to genetic mutations rather than CNV.

The tumor purity and ploidy of the samples were evaluated according to the CNV information of each tumor sample. In terms of genomic ploidy, the genomic ploidy of cluster 2 (3.9) was significantly lower than that of the other three clusters (5.17, 4.90, and 4.93 in cluster 1, 3 and 4, respectively, **Figure 5E**). The tumor purity of cluster 1 was lower than that of the other three clusters in terms of tumor purity (**Figure 5F**).

### Fusion Gene Characteristics in Subgroup

**Figure 6:**
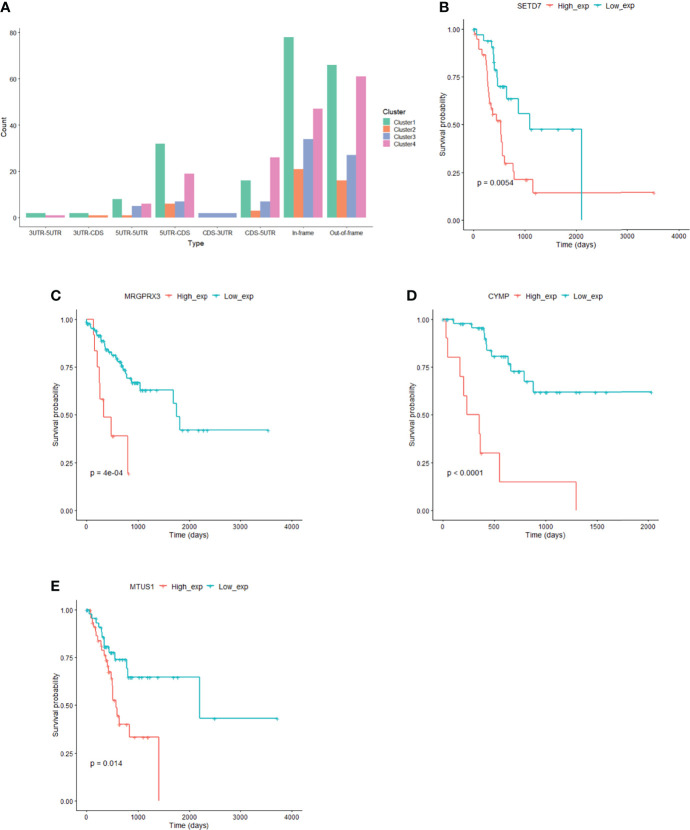
Fusion gene and prognostic markers in subgroups. **(A)** Fusion gene characteristics in subgroup. **(B)** SETD7 gene expression correlated with prognosis in cluster1. **(C)** MRGPRX3 gene expression correlated with prognosis in cluster2. **(D)** CYMP gene expression correlated with prognosis in cluster3. **(E)** MTUS1 gene expression correlated with prognosis in cluster4.

Based on the fusion genes found in the GC samples (**Supplementary DataSheet 5**), we analyzed the types of fusion genes in each subgroup. It was observed that the fusion of the CDS-3UTR type only occurred in cluster 3, and the fusion of the 3UTR-5UTR and 3UTR-CDS types only occurred in some clusters (**Figure 6A**), indicating that the types of fusion genes that occur among different subgroups are also different.

### Identification of Prognostic Markers in Subgroups

The expression of 110 genes in cluster 1, 45 genes in cluster 2, 89 genes in cluster 3, and 28 genes in cluster 4 significantly correlated with prognosis (P<0.05) (**Figures 6B–E**, **Supplementary DataSheet 6**). At the same time, we used Mut2SigCV to analyze driver genes based on the mutation data of the four subgroups. The results showed that seven genes (*RPL22*, *ZNF48*, *MAZ*, *CCDC38*, *MRGPRX3*, *LAMB4*, and *DNMT3L*) in subgroup 2 related to prognosis were also driver genes in subgroup 2.

### Driver Genes Correlated to GC Immune Infiltration

**Figure 7:**
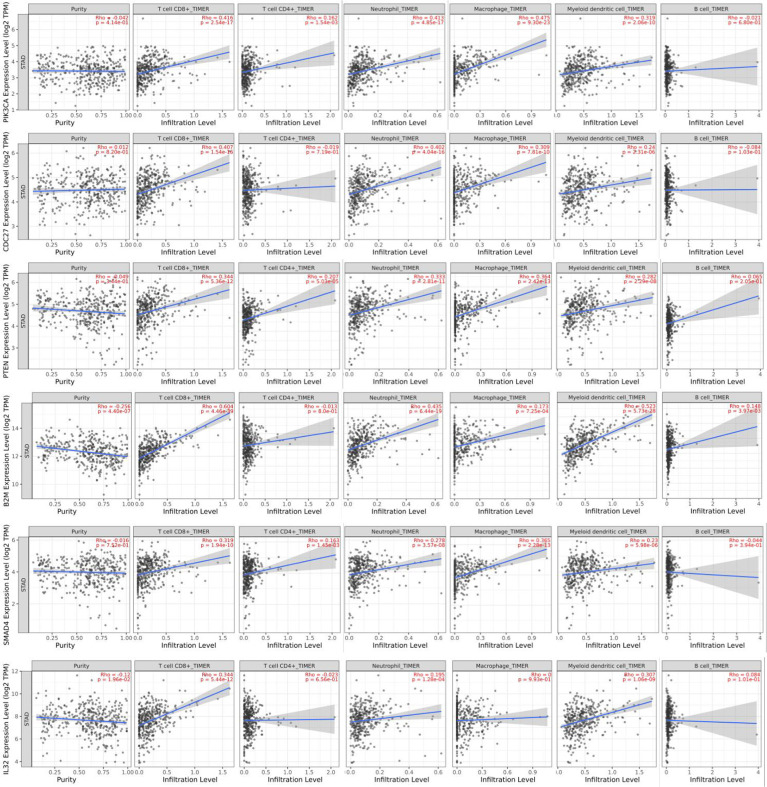
Correlation of six genes expression with immune infiltration level in GC.

Tumor-infiltrating lymphocytes are an independent risk factor for survival (8, 9). The researchers thus studied whether the pattern of immune infiltration was linked to the expression of candidate driver genes in GC. The analysis indicated that six genes (*PIK3CA*, *CDC27*, *B2M*, *PTEN*, *SMAD4*, and *IL32*) were strongly associated with tumor-infiltrating lymphocytes (**Figure 7**). Infiltrating levels of CD8+ T cells and DCs significantly correlated with the expressions of *PIK3CA*, *CDC27*, *B2M*, *PTEN*, SMAD4, and *IL32*. *PTEN* expression was associated with CD4+ T cell infiltration. The infiltrating levels of neutrophils significantly correlated with the expression of *PIK3CA*, *CDC27*, *B2M*, *PTEN*, and *SMAD4*. The infiltrating levels of macrophages significantly correlated with the expression of *PIK3CA*, *CDC27*, *PTEN*, and *SMAD4*. *B2M* expression strongly correlated with the infiltrating levels of B cells. These findings strongly suggest that *PIK3CA*, *CDC27*, *B2M*, *PTEN*, *SMAD4*, and *IL32* might play a critical role in the immune infiltration of GC, particularly CD8+ T cells and DCs.

### Correlation Analysis Between Driver Genes and Immune Marker Sets

To investigate the linkage between the six selected candidate genes and different immune infiltration cells, we investigated the correlations between the expression of the six selected genes and immune marker genes in various immune cell types, including CD8+ T cells, T cells in general, TAMs, M1 and M2 macrophages, neutrophils and DCs in GC (**Table 1**). In addition, different functional T cell populations, such as Th1 cells, T2 cells, Tregs, and exhausted T cells, were also analyzed. After correlation modification by purity, the results showed that the expression of the six selected genes corresponded considerably to most immune marker groups of various immune cells and T cells.

**Table 1:** Correlation analysis between related genes and markers of immune cells.

Description	Gene marker	PIK3CA		CDC27		B2M		PTEN		SMAD4		IL32	
		Cor	P	Cor	P	Cor	P	Cor	P	Cor	P	Cor	P
TAMs	CD163	0.529	***	0.506	***	0.454	***	0.365	***	0.444	***	0.252	***
	CSF1R	0.424	***	0.332	***	0.424	***	0.364	***	0.397	***	0.190	***
	FCGR2A	0.404	***	0.420	***	0.462	***	0.302	***	0.312	***	0.364	***
	IL10	0.380	***	0.391	***	0.387	***	0.335	***	0.345	***	0.165	**
	VSIG4	0.369	***	0.314	***	0.421	***	0.274	***	0.300	***	0.268	***
CD8+T	CD8A	0.248	***	0.207	***	0.529	***	0.251	***	0.189	***	0.355	***
	CD8B	0.173	***	0.137	**	0.341	***	0.081	0.114	0.121	0.018	0.253	***
Th1	CD4	0.355	***	0.288	***	0.500	***	0.280	***	0.307	***	0.288	***
	STAT4	0.437	***	0.362	***	0.478	***	0.342	***	0.344	***	0.319	***
	TBX21	0.252	***	0.254	***	0.474	***	0.277	***	0.208	***	0.340	***
Th2	CCR4	0.420	***	0.318	***	0.354	***	0.338	***	0.338	***	0.184	
	CCR8	0.437	***	0.394	***	0.437	***	0.296	***	0.308	***	0.351	
	CXCR4	0.383	***	0.260	***	0.326	***	0.277	***	0.277	***	0.073	
	GATA3	0.211	***	0.057	0.267	0.317	***	0.257	***	0.086	0.093	0.129	
Treg	CCR8	0.437	***	0.394	***	0.437	***	0.296	***	0.308	***	0.351	***
	FOXP3	0.254	***	0.219	***	0.393	***	0.165	**	0.220	***	0.327	***
	STAT5B	0.628	***	0.493	***	0.068	0.188	0.509	***	0.601	***	-0.06	0.019
	TGFB1	0.279	***	0.154	**	0.173	***	0.306	***	0.215	***	0.074	0.151
Neutrophils	CCR7	0.303	***	0.178	***	0.265	***	0.285	***	0.268	***	0.026	0.618
	CD66b	0.170	***	0.216	***	0.023	0.661	0.033	0.518	0.086	0.093	0.083	0.108
	CD11b	0.467	***	0.346	***	0.371	***	0.357	***	0.335	***	0.229	***
DCs	CD1c	0.264	***	0.064	0.217	0.165	**	0.273	***	0.208	***	-0.09	0.062
	ITGAX	0.449	***	0.403	***	0.419	***	0.323	***	0.320	***	0.311	***
	NRP1	0.541	***	0.378	***	0.260	***	0.377	***	0.362	***	0.065	0.203
M1	IRF5	0.284	***	0.236	***	0.201	***	0.194	***	0.174	***	0.059	0.255
	NOS2	0.035	0.5	0.145	**	0.146	**	0.056	0.277	0.107	*	0.286	***
	PTGS2	0.237	***	0.226	***	-0.01	0.985	0.064	0.217	0.172	***	0.114	*
M2	CCL2	0.109	0.3	0.053	0.3	0.195	***	0.138	**	0.09	0.08	0.105	0.04
	MS4A4A	0.416	***	0.351	***	0.506	***	0.329	***	0.324	***	0.278	***
exhausted T cell	CDC274	0.366	***	0.524	***	0.505	***	0.263	***	0.342	***	0.359	***
	CTLA4	0.296	***	0.328	***	0.412	***	0.194	***	0.289	***	0.313	***
	LAG3	0.118	***	0.164	***	0.525	***	0.129	0.012	0.115	0.025	0.426	***
	PDCD1	0.199	***	0.202	***	0.365	***	0.194	***	0.198	***	0.264	***
	TIGIT	0.354	***	0.329	***	0.479	***	0.287	***	0.305	***	0.350	***
	GZMB	0.072	0.1	0.215	***	0.536	***	0.027	0.6	0.042		0.552	***

These results further confirmed that the six selected genes were specifically associated with immune infiltration cells in GC, indicating that the six selected genes play an important role in the microenvironment of GC and immune escape.

### Pan-Cancer Analysis

**Figure 8:**
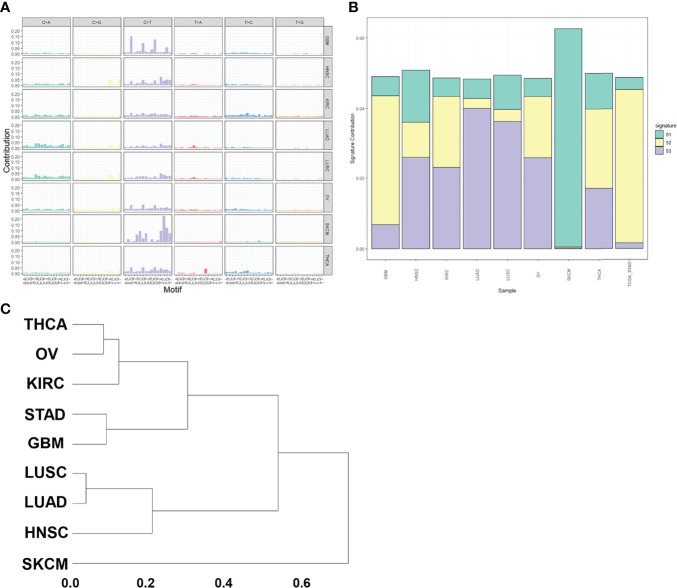
Pan-cancer analysis. **(A)** Mutation spectrum of pan-cancer samples. **(B)** Mutation feature ratio distribution. **(C)** Mutation feature clustering.

We selected the mutation data of the eight items of GBM, HNSC, KIRC, LUAD, LUSC, OV, SKCM and THCA in the pan-cancer analysis to analyze mutation characteristics. The mutation maps of these eight tumors are shown in **Figure 8A**. We then performed non-negative matrix factorization on these 8 tumor and GC mutation data to extract three mutation features. The contribution of each tumor to the three mutation features is shown in **Figure 8B**. Subsequent cluster analysis of the mutation spectrum from the analysis results can be seen that the mutation characteristics of GC and glioblastoma mutation characteristics were similar (**Figure 8C**). The similarity between the statistical mutation characteristics and the mutation characteristics collected using the Cosmic database can be used to determine which risk factors these mutation characteristics are related to. Our analysis was mainly related to the DNA mismatch repair system.

## Discussion

Continuously increasing genetic changes generally causes cancer. These include single nucleotide variations, small insertions or deletions, gene fusions, CNVs, and large chromosomal rearrangements. The most recent progress in sequencing technology has enabled scientists to generate large amounts of cancer genomes and categorize somatic mutations as rare and common types of cancer. To data, more than 10 major cancer categories have shown somatic mutations and specific characteristics. Nevertheless, finding driver mutations and cancer genes among millions of somatic mutations still represents an arduous task for humans.

There are many ways to classify mutations. Depending on whether they lead to cancer progression, driver and passenger mutation can be identified. The former has a selective growth benefit for tumor cells, while the latter does not directly or indirectly affect the selective growth benefit of tumor cells. MutSigCV could screen for genes with higher mutation frequencies, more mutations, and more mutations in preserved sites for high-frequency gene analysis of tumors. A total of 34 candidate driver genes were screened out, and three of them (*TP53*, *ARID1A*, and *PIK3CA*) were among the top20 most mutated. The screened candidates included previously known (*TP53*, *ARID1A* and *CDH1*) and new (*PIK3CA*, *CDC27*, *CTCF* and *IL12*, among others) significantly mutated driver genes. These driver genes are closely related to tumor prognosis. It has been reported that RPL22 controlled the dissemination of T-cell lymphoma (10). MAZ promoted prostate cancer bone metastasis by triggering transcriptional activation of the Kras-dependent RalGEFs pathway (11). DNMT3L is a novel marker and is essential for the growth of human carcinoma (12). LAMB4 is somatically mutated and expressionally altered in gastric and colorectal cancers (13).

We identified mutations in genes frequently mutated in GC. The study also revealed gene mutations linked to the cell migration and transcription. This demonstrates alterations in transcription and migration required for cancer progression, as illustrated in the latest spatial model of cancer development (14, 15). Mutational signatures in cancer have recently gained significant attention (16), and APOBEC-related signatures have been intensely investigated (17, 18). Gene mutation rates were significantly higher in APOBEC enrichment samples. According to earlier trials, the expression of APOBEC protein was linked to a poor prognosis of breast (19) and bladder cancer (17) and APOBEC mutational signatures have been observed at an increased rate over time in lung cancer (20). In this study, we found that APOBEC-related mutation characteristics are related to lymphocyte migration and cell-matrix adhesin, and these mutations could lead to tumor differentiation and adaptation under selective microenvironment from this subtype (21).

Tumor-infiltrating levels are independent predictors of survival in cancers. We identified six GC-related driver genes associated with the immune infiltration. It is revealed that high *B2M* expression was associated with elevated levels of CD8+T cells in GC and a high association with *B2M* expression in markers of CD8+T cells, such as CD8A and CD8B (**Table 1**). These results further revealed a relationship between *B2M* and CD8+T cell infiltration. Interestingly, *STAT5B*, a gene that regulates Treg cells, had a strong positive correlation with PIK3CA and SMAD4 expression (**Table 1**), suggesting that high PIK3CA and SMAD4 expression plays an important role in STAT5B mediating Treg cells. The six GC-related driver genes are also associated with the outcome and treatment response of immunotherapy. PIK3CA indicated an increasing risk of death in gastric cancer patients (22). B2M shapes the immune landscape of lung adenocarcinoma and causes resistance to PD-1-based immunotherapy (23). Loss of PTEN promotes resistance to T cell-mediated immunotherapy (24). IL-32 treatment reduced tumor growth and rendered immune checkpoint blockade-resistant melanoma responsive to anti–PD-1 therapy (25). Ablation of SMAD4 in tumor cells altered the immune TME and sensitized tumors to combination immunotherapy (26). Recurrent malignant gliomas patients with CDC27 mutations were more sensitive to immunoadjuvants and reirradiation therapy (27). And we have added this part into the discussion section.

Overall, our study provides several insights into the molecular basis of GC. We determined the significance of different in this disease. We also evaluated the influence of driving oncogenes in cancer pathogenesis and the link between immune infiltration and poor prognosis. The findings described in this study may play a role in further advancing GC diagnosis and treatment.

## Data Availability Statement

Publicly available datasets were analyzed in this study. This data can be found here: https://www.cancer.gov/about-nci/organization/ccg/research/structural-genomics/tcga.

## Ethics Statement

The studies involving human participants were reviewed and approved by Ethics Boards of Huashan Hospital of Fudan University. The patients/participants provided their written informed consent to participate in this study.

## Author Contributions

Conceptualization, ZZ; Investigation, YD, NS; Writing – Original Draft, YD, NS; Writing – Review & Editing, ZZ, JG, NS, LS, JW, JD; Visualization, ZZ; Supervision, JD; Funding Acquisition, JD, ZZ. All authors contributed to the article and approved the submitted version.

## Funding

This study was supported by the National Natural Science Foundation of China (grant No. 82002020 and No. 81270440).

## Conflict of Interest

The authors declare that the research was conducted in the absence of any commercial or financial relationships that could be construed as a potential conflict of interest.

## Publisher’s Note

All claims expressed in this article are solely those of the authors and do not necessarily represent those of their affiliated organizations, or those of the publisher, the editors and the reviewers. Any product that may be evaluated in this article, or claim that may be made by its manufacturer, is not guaranteed or endorsed by the publisher.

## Abbreviations

HNSC, Head and Neck squamous cell carcinoma; KIRC, Kidney renal clear cell carcinoma; LUAD, Lung adenocarcinoma; LUSC, Lung squamous cell carcinoma; OV, Ovarian serous cystadenocarcinoma; SKCM, Skin Cutaneous Melanoma; THCA, Thyroid carcinoma.
