# Supplementary material for: Driver Gene Alterations in Malignant Progression of Gastric Cancer
Source: Front Oncol. 2022 Jul 12;12:920207. doi: 10.3389/fonc.2022.920207 (PMC9315095; doi:10.3389/fonc.2022.920207)
Supplement: Supplementary file 1 [file Presentation_1.pdf]

Supplementary Material

Supplementary data

DataSheet 1: Differentially expressed genes in each subgroup.

DataSheet 2: Differentially expressed miRNAs in each subgroup.

DataSheet 3: The candidate driver genes of GC.

DataSheet 4: Different methylation sites of areas of differential expression in each subgroup.

DataSheet 5: The fusion genes in the GC samples.

DataSheet 6: The genes correlated with prognosis in each subgroup.

Supplementary Table1. Primer sequences used in the study

Gene	Forward primer	Reverse primer
ACVR2A	GTTTGCCGTCTTTCTTATCTCCT	GTCACCATAACACGGTTCAACA
ARID1A	CCAGCAGAACTCTCACGACC	CTGAGCGAAGGACGAAGACG
B2M	GAGGCTATCCAGCGTACTCCA	CGGCAGGCATACTCATCTTTT
C4orf26	AAGAAGAGGTATTTACGCCTCCT	GGTAGCGTCCGCATTATTTTGT
C7orf50	AAGCACAAGAACTGGAGGT	ATCGGGAACCTTGTCACTG
CBWD1	TTATTCAGAATCTCCTGTGGG	GTCTTTGATTGACACCAATCC
CDC27	CCCGTCCAGGCTGCTATATG	AAAGGCGTTCTGCGAGGAAAA
CDH1	ATTTTTCCCTCGACACCCGAT	TCCCAGGCGTAGACCAAGA
CDKN2A	GAAGGTCCCTCAGACATCC	GTAGGACCTTCGGTGACTG
CTCF	CAGTGGAGAATTGGTTCGGCA	CTGGCGTAATCGCACATGGA
HRCT1	GAGAGGAACCGTACAGCTG	GTGAAAGATTCCCAGGTGG
IL32	TGGCGGCTTATTATGAGGAGC	CTCGGCACCGTAATCCATCTC
KLF3	CCTGGGATCGAACCACAGAG	CCGGGGATTCCACAGGTAAAG
KRAS	GAGTGCCTTGACGATACAG	TGCTTCCTGTAGGAATCCTC
LARP4B	GACAAGTTCCATCCCACCTTTG	CCATTGGCATCCGATCCCT
MAP2K7	GGGACGTTCATCACCAACAC	GCCACTGTCATCTTGCCCA
MUC6	CTGCCCTATACCAGCAATGGA	CTGACCCATGTACTTCCGCTC
MXRA8	GCAACCTGCACCATCACTACT	CCACCTGTTGAGCCTCCTC
NUDT11	GAGAAACTAAAGCTGGGCG	GCTGTTCATTAGGGATCGC
OR5M3	TTCACCGATGTGACAGAGTTCA	CATGCCGATATTGCCCACCAT
PIK3CA	CCACGACCATCATCAGGTGAA	CCTCACGGAGGCATTCTAAAGT
POLDIP2	TGCTGATTGATGCTCGTGACT	CCTGGGATGGCATAGAGGG
PTEN	TTTGAAGACCATAACCCACCAC	ATTACACCAGTTCGTCCCTTTC
PTH2	CTCCACAGGTGATGGAGAC	TGTACGAGTTCAGCCAGTG
RHOA	GGAAAGCAGGTAGAGTTGGCT	GGCTGTCGATGGAAAAACACAT
RNF43	GGTGTTGATCTGGGGTAATGAC	CAATCCTCACATGGGCCTTTT
RPL22	AAAGTGAACGGAAAAGCTGGG	TCACGGTGATCTTGCTCTTGC
SMAD4	CTCATGTGATCTATGCCCGTC	AGGTGATACAACTCGTTCGTAGT
SMAP1	ACCAGCTCATCCTATCCAAGC	GAAGCCCATCGAGGACCTT
SNRNP27	CAGGTCTCGGGAGAGAGATCG	TGTCGTCTTGGAGATCGGGAG
TCERG1	CCTCCGGGTATGATGTTTCCA	CAACCCATATCTCCTCCGTAGG
TP53	CAGCACATGACGGAGGTTGT	TCATCCAAATACTCCACACGC
TTK	GTGGAGCAGTACCACTAGAAATG	CCCAAGTGAACCGGAAAATGA
XYLT2	AGGTGGTACGGGCAGTAAC	GCTCCCTGTATCTCCGTGT

Supplemental Figure 1

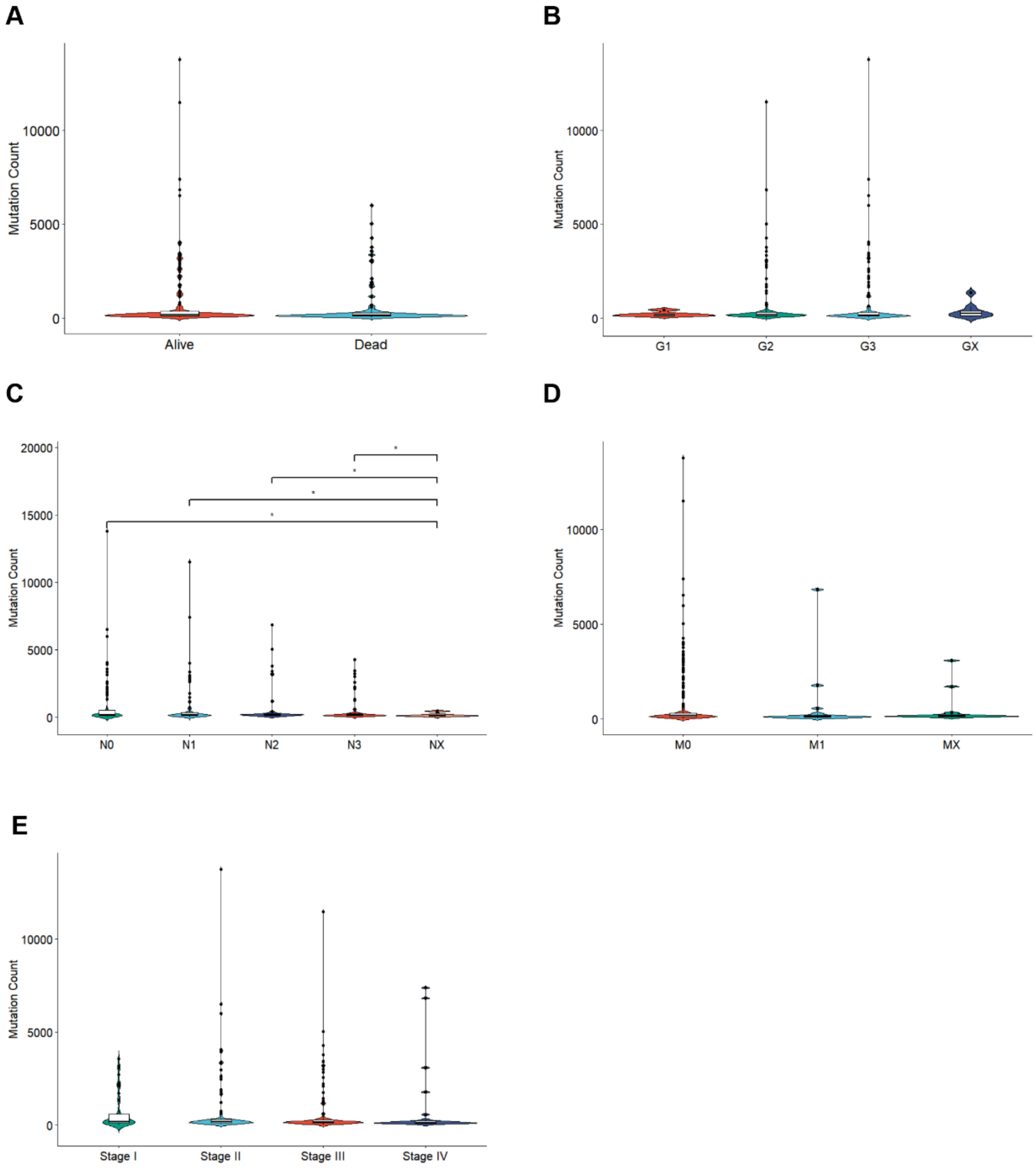

Supplemental Figure 1: Correlation between mutations and clinical features. (A) Correlation between mutations and status. (B) Correlation between mutations and grades. (C) Correlation between mutations and primary tumor. (D) Correlation between mutations and lymphatic metastasis. (E) Correlation between mutations and distant metastasis. (F) Correlation between mutations and staging.

Supplemental Figure 2

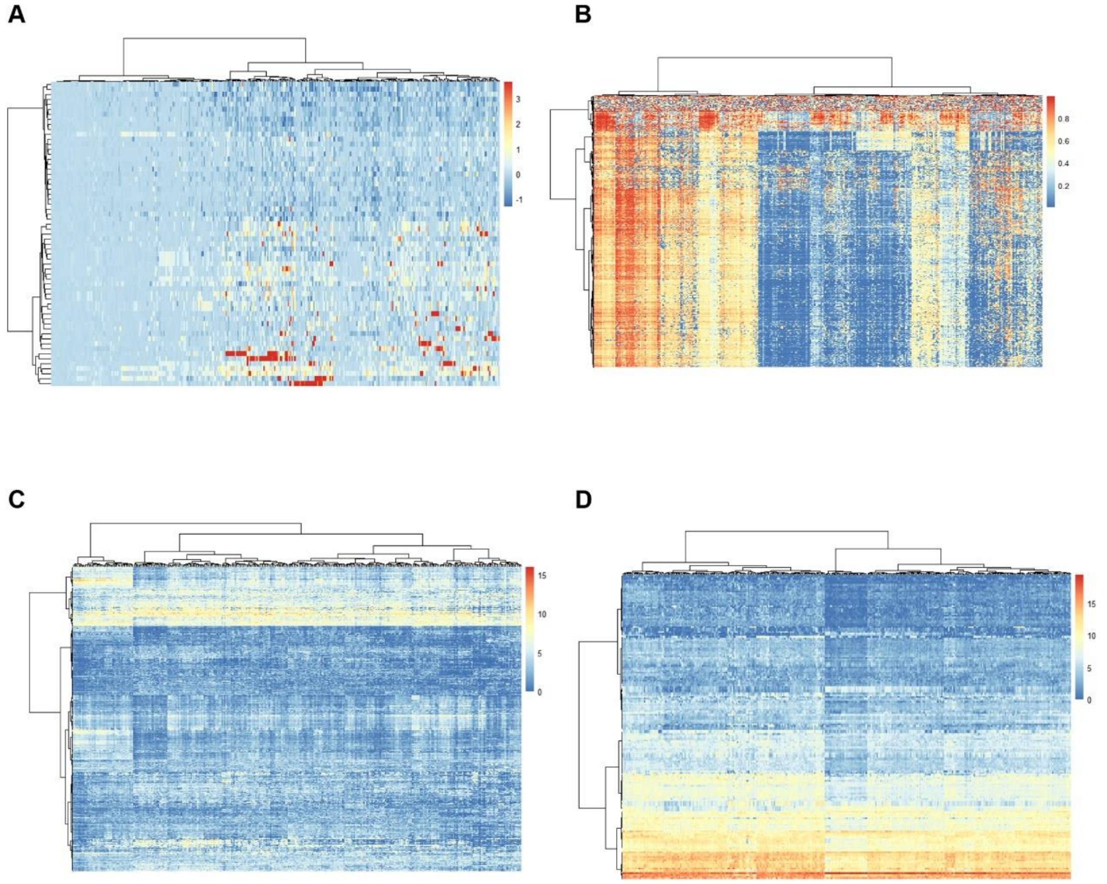

Supplemental Figure 2: Data clustering on different data platforms. (A) CNV clustering results. (B) Methylation expression profile clustering results. (C) mRNA expression profile clustering results. (D) miRNA expression profile clustering results.

Supplemental Figure 3

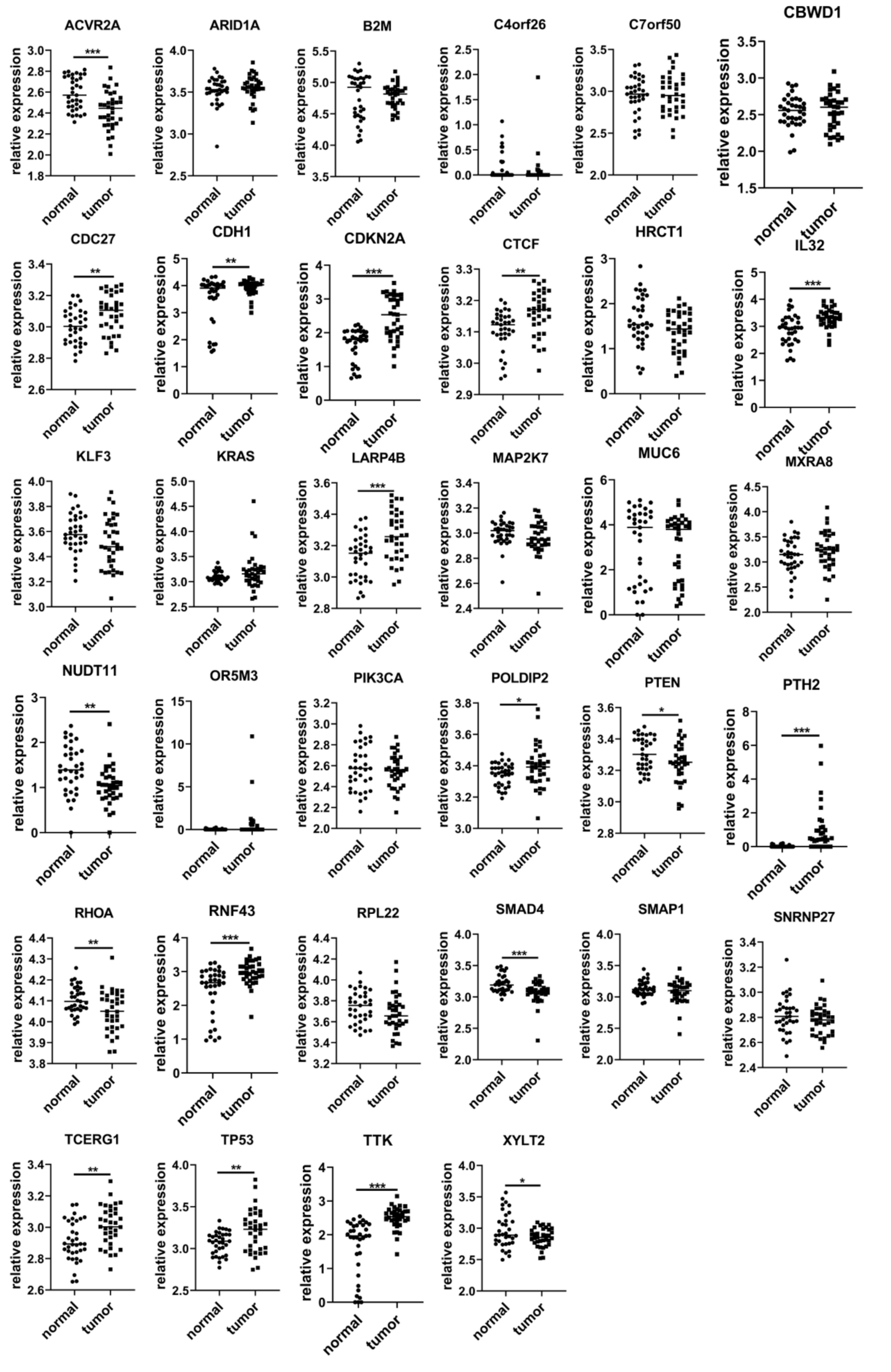

Supplemental Figure 3: mRNA expression of driver genes. mRNA of driver genes

was detected by PCR in both tumor and normal tissues (n=30). Data are represented as mean ± SD. Asterisks indicate statistically significant differences from each other; *p < 0.05, **p < 0.01, ***p < 0.001.

Supplemental Figure 4

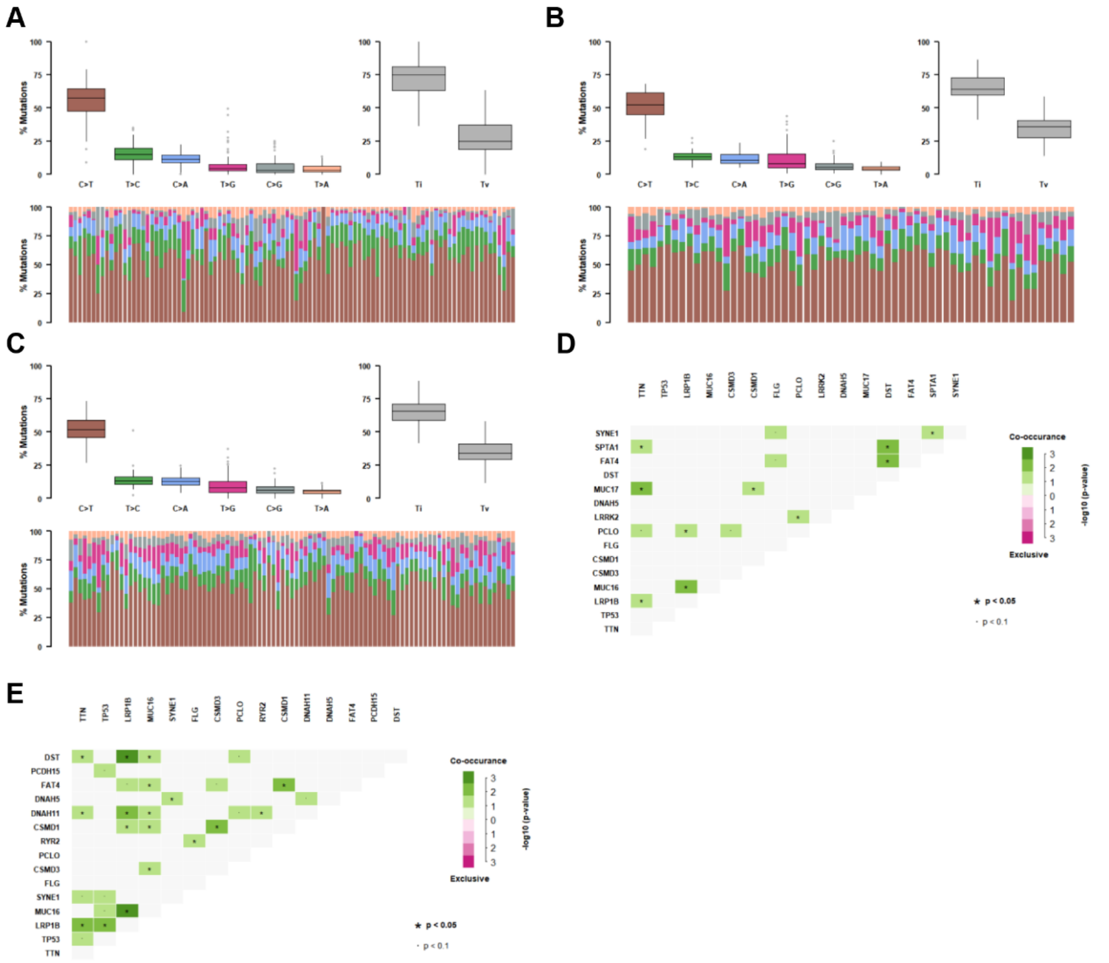

Supplemental Figure 4: Mutation in subgroups. (A) Mutation type distribution in cluster 2. (B) Mutation type distribution in cluster 3. (C) Mutation type distribution in cluster 4. (D) Co-occurrence and mutual exclusivity of genetic alterations in cluster 3. (E) Co-occurrence and mutual exclusivity of genetic alterations in cluster 4.

Supplemental Figure 5

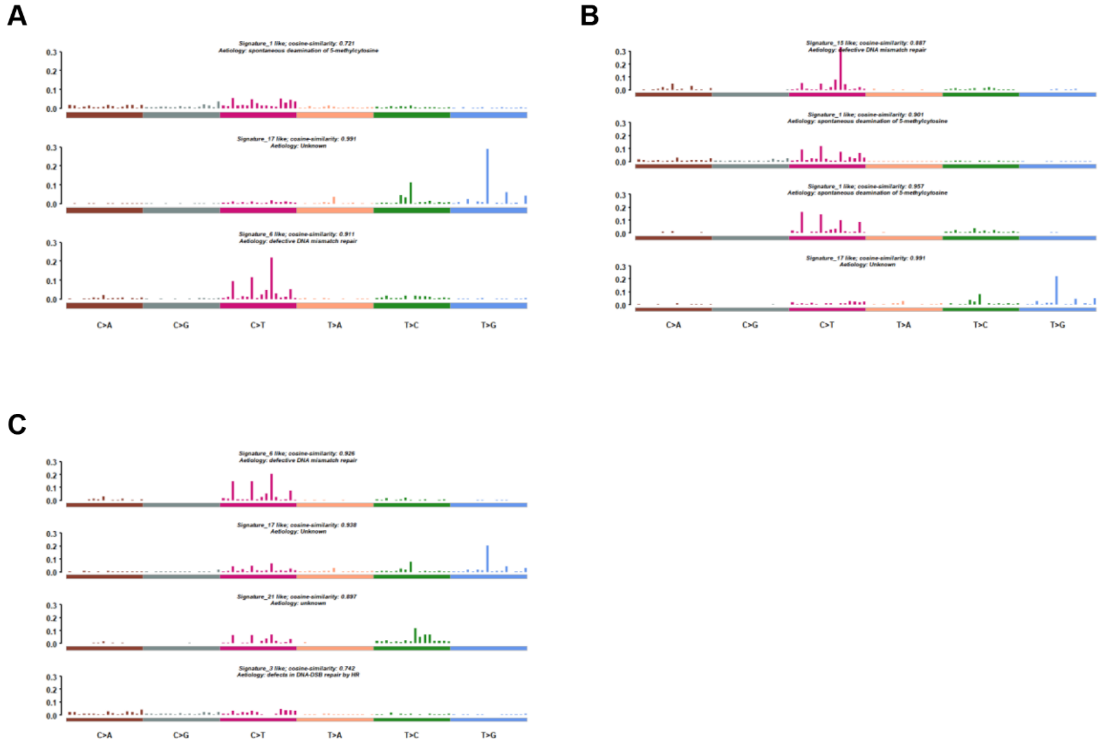

Supplemental Figure 5: The similarity analysis of signatures in subgroups and cosmic signature. (A) The signature with high degree similarity in cluster 1 and cosmic signature. (B) The signature with high degree similarity in cluster 3 and cosmic signature. (C) The signature with high degree similarity in cluster 4 and cosmic signature.
